# Hollow-core fibres for temperature-insensitive fibre optics and its demonstration in an Optoelectronic oscillator

**DOI:** 10.1038/s41598-018-36064-1

**Published:** 2018-12-20

**Authors:** U. S. Mutugala, E. R. Numkam Fokoua, Y. Chen, T. Bradley, S. R. Sandoghchi, G. T. Jasion, R. Curtis, M. N. Petrovich, F. Poletti, D. J. Richardson, R. Slavík

**Affiliations:** Optoelectronics Research Centre, University of Southampton, Southampton, SO17 1BJ UK

## Abstract

Many scientific and practical applications require the propagation time through cables to be well defined and known, e.g., an error in the evaluation of signal propagation time in the OPERA experiment in 2011 initially erroneously concluded that Neutrinos are faster than light. In fact, there are many other physical infrastructures such as synchrotrons, particle accelerators, telescope arrays and phase arrayed antennae that also rely on precise time synchronization. Time synchronization is also of importance in new practical applications like autonomous manufacturing (e.g., synchronization of assembly line robots) and upcoming 5G networks. Even when the propagation time through a coaxial cable or optical fibre is carefully calibrated, it is affected by changes in the ambient temperature, posing a serious technological challenge. We show how hollow-core optical fibres can address this issue.

## Introduction

**Figure 1:**
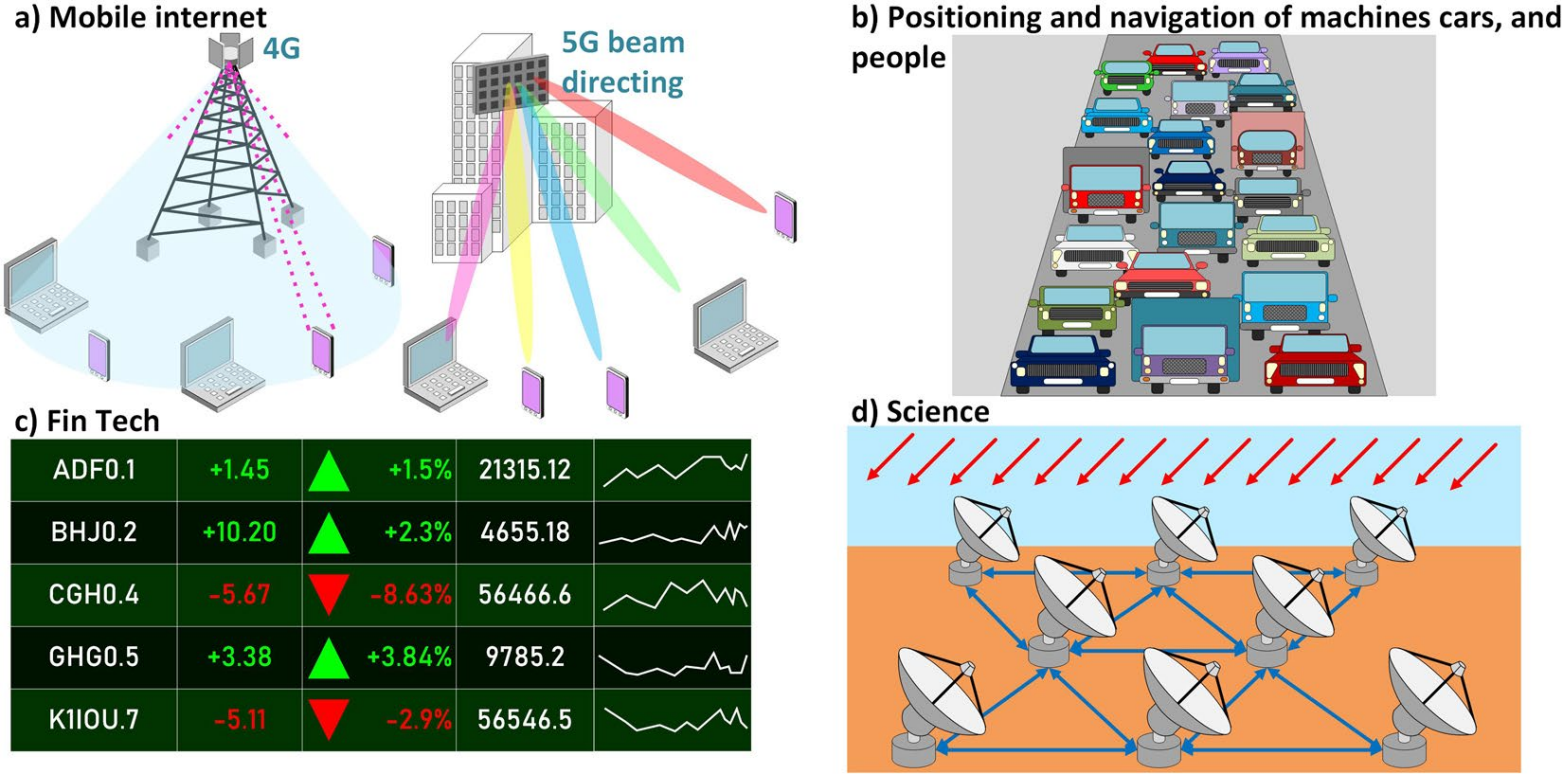
Examples of applications in which better timing/synchronization than currently available is important. (**a**) Emerging 5G networks: significantly higher capacity will be achieved by sending information only in the direction of the user. To do so, the precise position of the user must be known. To steer the signal direction, tight synchronization amongst a large array of antennae is needed. (**b**) For precise coordination of cars and machines that work together, tight synchronization is needed. (**c**) Financial markets, and also data centres rely on precise synchronization to establish order of the transactions. (**d**) Signal from widely-spaced telescopes need to be precisely time managed to establish the direction of the incoming signal.

**Figure 2:**
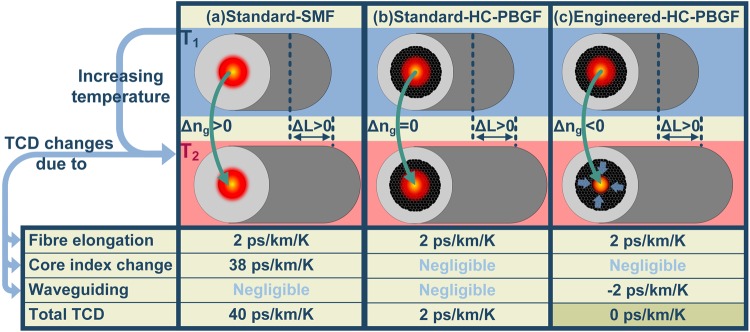
Thermal sensitivity (TCD) of various types of fibres. (**a**) In standard fibre the TCD is dominated by the thermo-optic coefficient of the glass material in the fibre core through which the light propagates. (**b**) This is eliminated in HCF (e.g., HC-PBGFs) in which light propagates through air, making thermally-induced fibre elongation the dominant effect that determines the TCD. (**c**) In engineered HC-PBGF, changes in the waveguide properties (e.g., a larger fraction of the guided mode is inside the central hollow fibre core as the fibre is heated up and the transverse fibre structure expands) can be set to have a contribution to the TCD that has the opposite sign and equal magnitude contribution to the fibre elongation, making HC-PBGF thermally insensitive. n_g_: group index; ΔL: change of length; HC-PBGF: Hollow Core Photonics Bandgap Fiber; SMF: single-mode fiber.

Examples of applications in which better timing/synchronization than currently available is important are shown in Fig. 1. The thermal sensitivity of any signal-transmitting medium is determined by two factors: its elongation with temperature and the change of propagation speed with temperature. Thanks to the extremely low coefficient of thermal expansion of silica glass (5 × 10^−7^/K), optical fibres are one of the least thermally-expanding materials, making optical fibres good candiates for thermally-insensitive applications. Unfortunatelly, the thermo-optic coefficient of silica glass is moderate, reducing the benefit of the low thermal expansion significantly^1^, Fig. 2. As a result, although the total rate of change of propagation time through a conventional optical fibre with temperature (thermal coefficient of delay, TCD ~40 ps/km/K^1^) is generally lower than in coaxial cables^2,3^, it cannot be ignored in most applications. The adverse effect of the silica glass thermo-optic coefficent on the fibre TCD can fortunately be strongly suppressed in hollow core optical fibres (HCF), in which the light propagates through a hollow core rather than a (thermally-sensitive) silica glass core, Fig. 2. This makes HCFs one of the least thermally-sensitive transmission media with a TCD almost 20 times smaller than standard silica fibre (~2 ps/km/K^4,5^), Fig. 2. This was first demonstrated on small lengths of HCF^4^ and recently on a 860-m length in an application demonstrator which used HCF as a delay line in an Optoelectronic Oscillator, OEO^5^.

Although HCF has already been demonstrated to be an excellent solution for stable signal propagation, we predicted that its thermal sensitivity can be further lowered and even completely eliminated^6^. This intriguing property can be achieved in HCFs that rely on a photonic bandgap for light guidance, known as hollow core photonic bandgap fibres (HC-PBGF)^7^. We predicted^6^ that when heating the fibre, the mode slows down on the short wavelength edge of the bandgap transmission window and speeds up at its long wavelength edge. This gives an opportunity to compensate for the (already small) temperature-induced HC-PBGF elongation by making the mode travel faster at the bandgap long wavelength edge, Fig. 2. We carried out a preliminary proof-of-principle experiment^6^ in which we demonstrated the predicted unique property of zero TCD in a short piece (2.8 m) of HC-PBGF. Although these preliminary results were encouraging, they were obtained using a 7-cell geometry^8^ that suffers from relatively high propagation loss (~10 dB/km^8^); relied on an indirect measurement (measuring spectral phase and calculating delay as its derivative); and were made for a short length of fibre. However, most practical uses of zero TCD fibre will require significantly longer lengths (100 s of meters to km and beyond (e.g. for 5 G networks, time synchronization in large data centres, delay-line stabilized ultra-stable lasers for metrology^9^, etc.), thereby requiring a different HC-PBGF design offering significantly lower losses. Indeed, demonstration over a significantly longer length (requiring sufficiently uniform fibre along its lengths) is still to be shown. A demonstration of a low loss zero TCD fibre in an application is another necessary step to make this approach appealing to the wider scientific and engineering communities.

Here we demonstrate that the technology of HC-PBGF with zero TCD has matured sufficiently to be used in many of the applications envisaged. To achieve this, we have made several key improvements. We have used a practical fibre design (19-cell) that has previously yielded the lowest reported losses of 1.7 dB/km^10^ and the longest manufacturable lengths (>10 km^11^). We show that this fibre geometry can provide zero TCD over practical fibre lengths (>1 km) for the first time, and demonstrate its use and performance in an application (thermally-insensitive OEO). Furthermore, near zero thermal sensitivity is measured directly since the frequency of the OEO signal is directly related to the delay of the fibre delay line. Measurements (see Supplementary Information) suggest good longitudinal uniformity of the fibre thermal sensitivity along its length.

## Results

**Figure 3:**
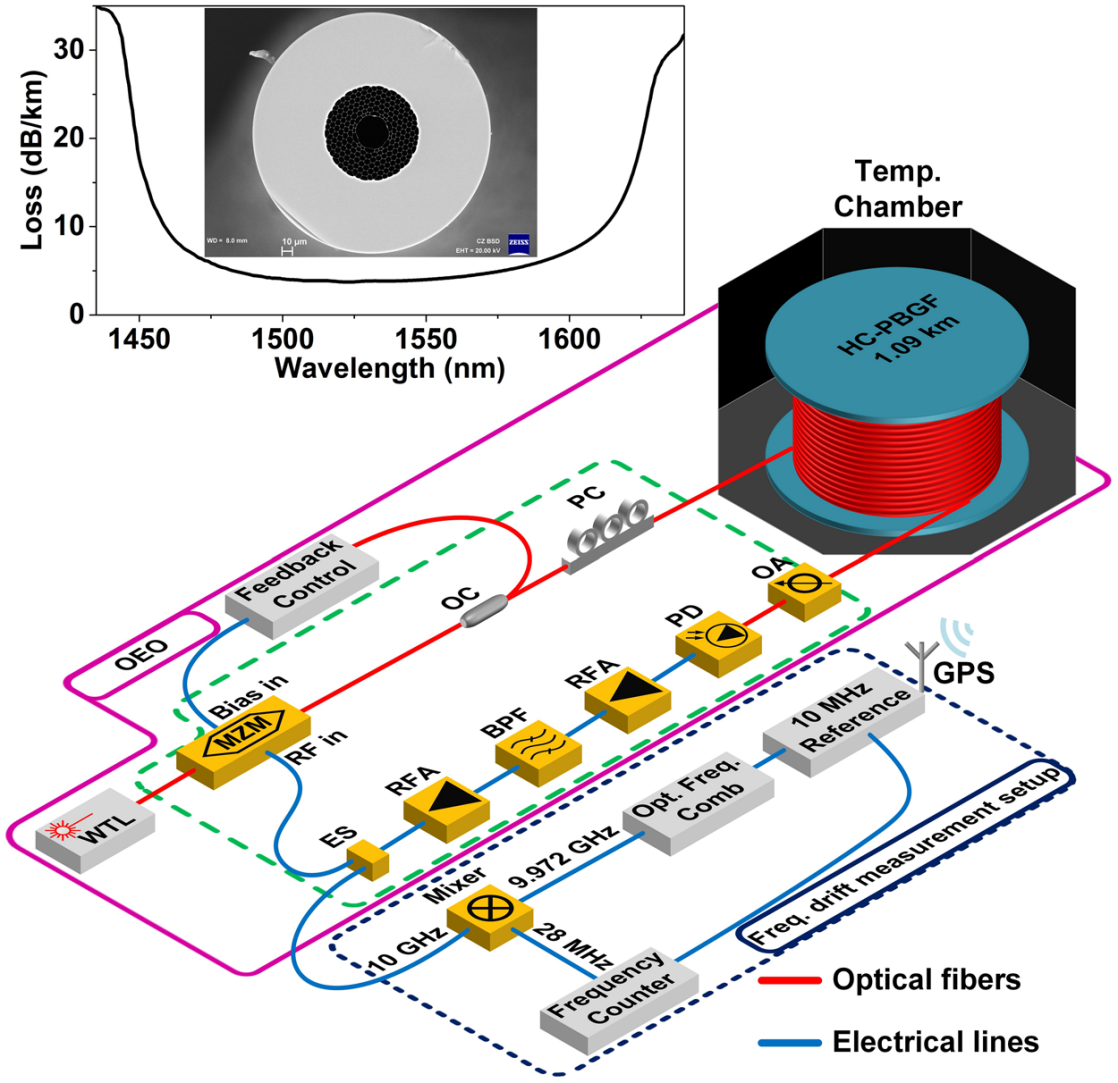
Experimental setup of the OEO. The oscillator cavity consists of the length of HC-PBGF (end face and spectral transmission characteristics shown in inset) placed in a thermally-controlled chamber and with the remaining components (inside the green-dashed-border) being thermally shielded. The blue-dot-bordered part is dedicated to generating a stable 9.972-GHz RF signal reference and using it to measure the OEO frequency drift. WTL: Wavelength-tunable laser; MZM: Mach-Zehnder amplitude modulator, OC: Optical coupler; PC: Polarization controller; OA: Optical Attenuator; PD: Photodiode; RFA: RF amplification; BPF: Band-pass filter, ES: RF splitter; GPS: Global positioning system.

Our fibre was 1.09 km long with a 31 µm core diameter, Fig. 3. The fibre loss was 4 dB/km at 1550 nm and 9.5 dB/km at 1610 nm where our simulations predicted the zero TCD would be achieved. The fibre was spliced to standard SMF-28 pigtails (with a splice loss of ~1 dB/splice) to enable it to be interfaced to standard fibre components.

It is worth mentioning that this pigtailed fibre sample (6.1 dB loss) is significantly better than that which we used previously (15 dB loss^5^) in a prior OEO demonstration which benefited only from the reduced TCD of the HC-PBF (rather than the complete thermal insensitivity presented here).

To illustrate and quantfy the application benefits of using zero TCD HC-PBGFs and to allow comparitive measurements against standard fibre in an application we chose to focus on the case of an OEO, which requires extremely high levels of fibre performance. The OEO^12,13^, is an oscillator in which the resonating signal propagates as a modulated optical carrier in one part of the cavity and as a radio-frequency (RF) signal in the other. It produces high-frequency (10 s of GHz) RF signals of extremely high purity as needed for example in metrology, radio astronomy, radar, communications, etc. To achieve extremely low noise in the oscillator, a long optical delay (e.g., using a km-length optical fibre) is needed. However, as mentioned earlier, the delay imparted by the optical delay line usually changes with temperature (e.g., in a 1-km standard optical fibre, at a rate of 40 ps/K), introducing undesirable drift into the OEO oscillating frequency. An optical delay line with zero TCD could in principle completely eliminate this unwanted drift.

**Figure 4:**
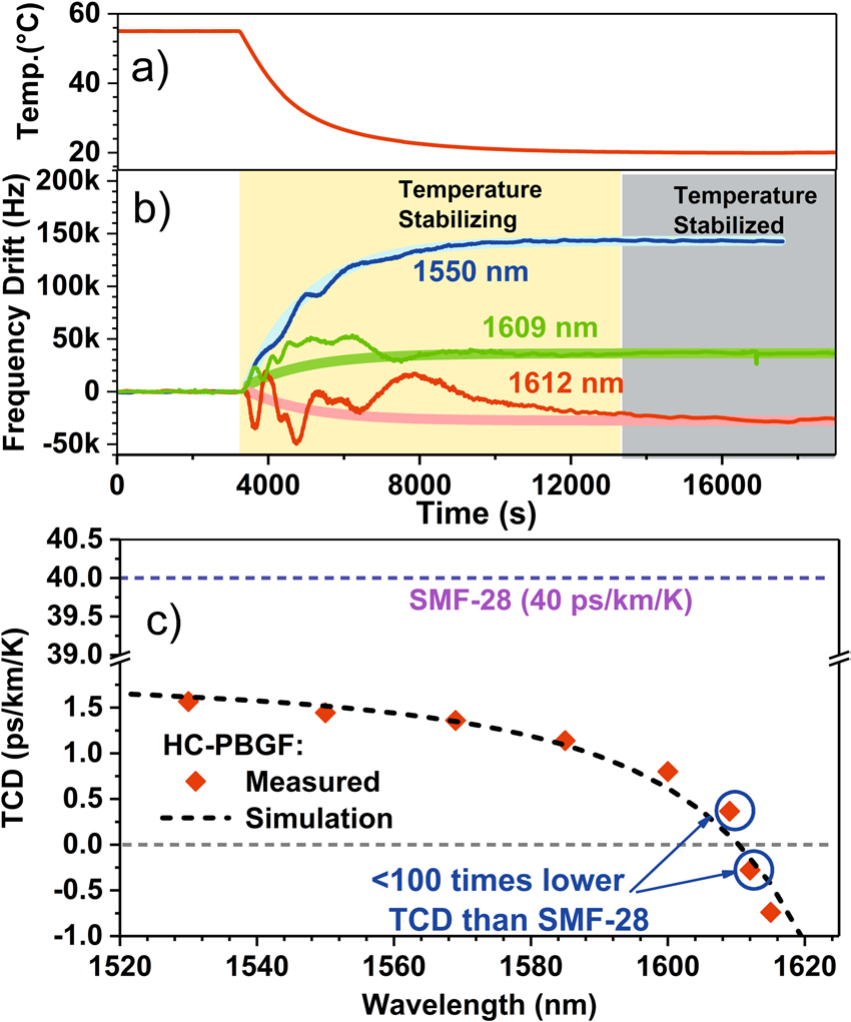
Fibre temperature (**a**) and corresponding OEO oscillating frequency change (**b**) for three selected laser wavelengths of 1550 nm (positive change), 1609 nm (close-to-zero, but still a positive change) and 1612 nm (close-to-zero, but a negative change). The expected change (from our simulations) is also shown. During the ‘temperature stabilizing’ time period, the temperature inside the thermal chamber does not change uniformly across the chamber, producing ripples in the measured curve. The results shown in (**b**) were used to evaluate the HC-PBGF TCD (**c**), shown together with our prediction based on simulations. For comparison, the TCD of SMF-28^4,5^ is also shown.

Our OEO is shown in Fig. 3. OEO frequency drifts due to temperature change (from 55 °C to 20 °C, Fig. 4a) were measured by comparing the signal with that of a stable reference derived from an Optical Frequency Comb. As the thermal properties of the HC-PBGF are expected to depend on the wavelength of the optical signal propagating through it^6^, we repeated the experiment at various wavelengths between 1530 nm and 1615 nm. Details of the setup are given in the Methods section. Our results are shown in Fig. 4. In Fig. 4b we see that the frequency drift and thus the change of the propagation time through the HC-PBGF can be positive or negative, depending on the wavelength of the signal, and which crosses zero (zero TCD point) around 1610 nm, Fig. 4c. These results agree well with predictions made using the simplified model we published in^6^, Fig. 4c.

The HC-PBGF TCD was measured to be 0.37 ps/km/K at 1609 nm and −0.28 ps/km/K at 1612 nm. This shows even though the zero-TCD behavior occurs at one specific wavelength, there is a >3 nm wavelength window within which the HC-PBGF TCD is ~100 times lower than that of SMF-28. We have not measured at the zero-sensitivity wavelength itself, as the HC-PBGF sensitivity was too low there to be reliably measured due to the small residual drifts associated with the other OEO components.

We also measured the HC-PBGF chromatic dispersion properties (on a short piece, as well as along the entire length), which were predicted^6^ to be critical in defining the zero-thermal sensitivity of these fibres. These results are presented in the Supplementary Information.

**Figure 5:**
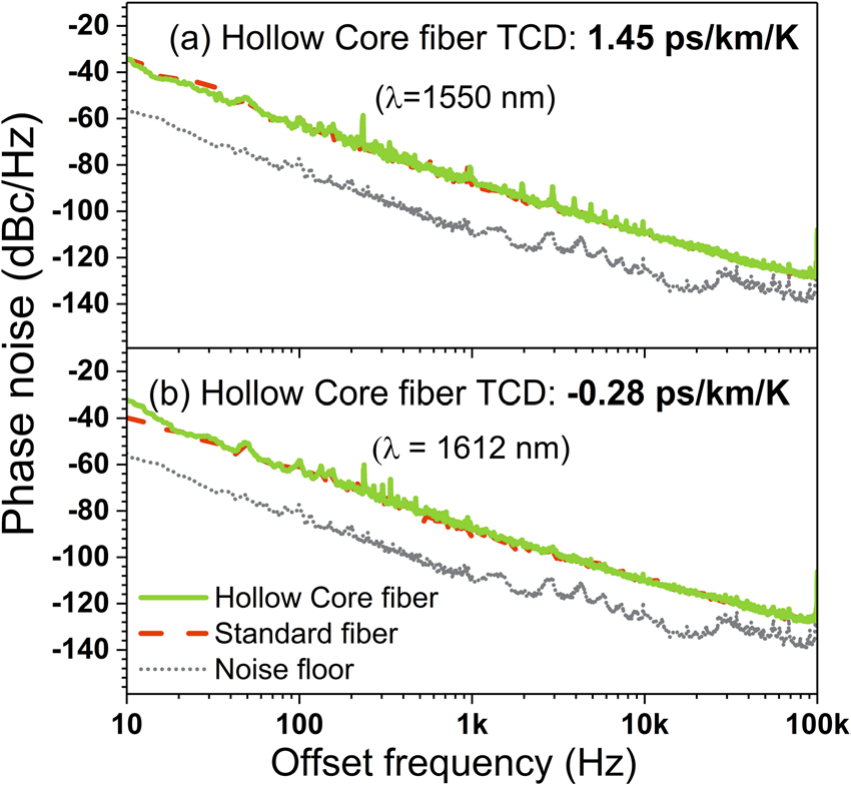
Phase noise properties of the OEO. (**a**) at 1550 nm and (**b**) 1612 nm operating wavelengths when the HC-PBGF was used as used as a delay line (green) and when a standard optical fibre that introduced a similar delay was used as a delay line (dashed-red) for comparison. No degradation of the noise performance is seen, showing stable performance of the HC-PBGF based system (which offers a >100-fold reduced temperature sensitivity).

Finally, Fig. 5 shows the phase noise of the OEO with the HC-PBGF at two operating wavelengths of 1550 nm (HC-PBGF TCD is positive) and 1612 nm (HC-PBGF TCD is very small and slightly negative). For comparison the phase noise measured using a 700 m long length of SMF-28^14^ (which induced a similar delay to that of the HC-PBGF, 3.43 µs) is also shown, showing there is no measurable noise penalty in using HC-PBGF as compared to standard fibre.

## Conclusions

In this work we demonstrated for the first time long-length (>1 km), low-loss (minimum loss of 4 dB/km) HC-PBGF with zero thermal delay sensitivity and its use in a demanding application. The approach opens the door to significant performance improvement in many emerging applications (some outlined in Fig. 1), most of them requiring fibre length and loss levels that we have already achieved. Besides the zero thermal sensitivy, HCFs have other unique and enabling properties including low latency, low non-linear signal distortions, immunity to radiation, and the ability to transmit very high laser powers.

## Methods

### OEO Setup

A wavelength tunable (1520 nm to 1630 nm) continuous-wave laser (Agilent) is used as the light source of the OEO. Laser light was propagated through a LiNbO_3_ Mach-Zehnder modulator (MZM). MZM output was launched into the HC-PBGF delay line. Since splices between HC-PBGF-to-SMF pigtails introduced a slight polarization dependent loss (PDL) which was measured to be ~0.3 dB, a polarization controller was used at the input of the HC-PBGF. The high-speed photodiode (PD) connected to the output of the HC-PBGF converts the optical signal into an electrical signal. Since the transmission loss of HC-PBGF is wavelength dependent, a variable optical attenuator was used before the PD to maintain −6 dBm optical power at the PD for phase noise measurements. The electrical signal was amplified by low noise amplifiers (ultra-low phase noise amplifier with 14 dB gain and low noise amplifier with 17 dB) and filtered by a 10 GHz electrical band-pass filter (with 8 MHz 3 dB bandwidth). The filtered 10 GHz electrical signal was then re-amplified (low noise amplifier with 17 dB gain and high power amplifier with 37 dB gain). The amplified electrical signal was split into two: one used to close the cavity by feeding it back to the MZM, while the other part serves as the OEO output. MZM was maintained at quadrature biased position using a feedback control loop^15^. The HC-PBGF delay line was kept inside a thermal chamber that had omnidirectional temperature control (heating elements placed on all sides) while the rest of the OEO components were kept shielded from ambient temperature variations by covering with insulation foam.

Intensity noise of light propagating through a HC-PBGF is increased due to multi-path interference^16^, leading to the degradation of phase noise in OEOs^17^. To supress this, frequency modulation of the laser was used^16,17^ via in-built modulation (coherence control) of the used laser.

### Frequency drift measurement setup

The reference signal at 9.972 GHz frequency was generated by detecting the beating between tones of an optical frequency comb (Menlo Systems) with a repetition rate of 249.3 MHz with a PD and filtering out the 40^th^ RF tone. The OEO signal and the reference signal were fed into a mixer and the down converted mixer output at 28 MHz was measured with a frequency counter (Menlo Systems). The frequency counter and the repetition rate of the frequency comb were kept synchronized to a highly stable 10 MHz oscillator (Timetech) which was locked to a Global Positioning System (GPS) signal.

### TCD calculations

A change of propagation time through the HC-PBGF changes the OEO cavity delay (the rest of the OEO components are shielded from ambient temperature variations). This changes the free spectral range (FSR) of the OEO cavity. The change of FSR can be observed as a change of the carrier frequency of the OEO. If the OEO cavity delays at two different temperatures are given by τ_1_ and τ_2_ and the corresponding FSRs are FSR_1_ and FSR_2_, by assuming FSR≈FSR_1_≈FSR_2_, the change of propagation time through the whole length of HC-PBGF (Δτ) can be given by (1) where *f*_*drift*_ is the change of carrier frequency and *f*_*osc*_ is the carrier frequency^5^. The FSR of the OEO cavity, which is the mode spacing between the OEO carrier frequency and the spurious tones was measured to be 260 kHz.

1 $${\rm{\Delta }}{\rm{\tau }}={{\rm{\tau }}}_{2}-{{\rm{\tau }}}_{1}=\frac{1}{{{\rm{FSR}}}_{2}}-\frac{1}{{{\rm{FSR}}}_{1}}\approx \frac{{\rm{\Delta }}\mathrm{FSR}}{{{\rm{FSR}}}^{2}}=\frac{{{\rm{f}}}_{{\rm{drift}}}}{{{\rm{f}}}_{{\rm{osc}}}{\rm{FSR}}}$$

The TCD was then calculated according to^5^,

2 $${\rm{TCD}}=\frac{1}{{\rm{\Delta }}\mathrm{TL}}[\frac{{{\rm{f}}}_{{\rm{drift}}}}{{{\rm{f}}}_{{\rm{osc}}}{\rm{FSR}}}]$$

where ΔT is the temperature change and L is fibre length.

### FEM simulations

The simulated TCD was obtained as follows: A permittivity profile was obtained from a high resolution scanning electron microscope image of the fiber cross-section using the method detailed in^18^. Finite element calculations of modal properties such as effective index and fractional field intensity carried in glass were performed on this fiber profile using the commercial software COMSOL Multiphysics. The TCD was finally obtained by simply evaluating the expressions given in ref.^6^, also taking into account the thermal effects of the single acrylate coating layer protecting the fiber. The simulation results shown as the dashed line in Fig. 4c predicted a zero crossing at 1610.3 nm, in remarkably good agreement with our measurements.

### Phase noise measurement

Photonic delay line technique^19^ was used for measuring phase noise. A laser (Orbits Lightwave Inc., USA) operating with 18 dBm output power was used in the setup. Two fiber delay-lines; 1 km and 5.2 km were used in the phase noise measurement setup and the measurements were stitched together. Noise floor was measured by removing the delay-lines.

## Electronic supplementary material

Supplementary Information
