# Supplementary material for: Hollow-core fibres for temperature-insensitive fibre optics and its demonstration in an Optoelectronic oscillator
Source: Sci Rep. 2018 Dec 20;8:18015. doi: 10.1038/s41598-018-36064-1 (PMC6302091; doi:10.1038/s41598-018-36064-1)
Supplement: Supplementary file 1 — Supplementary Information [file 41598_2018_36064_MOESM1_ESM.docx]

Hollow-core fibres for temperature-insensitive fibre optics and its demonstration in an Optoelectronic oscillator

U.S. Mutugala, E.R. Numkam Fokoua, Y. Chen, T. Bradley, S. R. Sandoghchi, G.T. Jasion, R. Curtis, M.N. Petrovich, F. Poletti, D.J. Richardson, and R. Slavík

**Supplementary information**

In reference [1], it was predicted that there is a relationship between the HC-PBGF zero thermal sensitivity and its chromatic dispersion (CD). This observation is interesting from a practical point of view: it is significantly easier and quicker to measure fibre CD than the thermally-induced propagation delay changes, which are very small for hollow core fibre. Thus, we first simulated and measured the CD of the HC-PBGF to identify its predicted zero thermal sensitivity wavelength – to confirm it is within wavelength of our source. Another practical consideration is the hollow core fibre loss at the zero-thermal sensitivity point – for low loss, it must lie at a position within the photonic bandgap where the transmission loss is low.

A simplified analysis of the contributions to thermal sensitivity in HC-PBGFs predicts that a CD = 79 ps/nm/km corresponds approximately to the zero thermal sensitivity point. However, this is for a bare fiber (with no coating) and the value is expected to change slightly when a fiber with a polymer coating is considered [1].

Finite element simulations of the CD from an SEM image of our HC-PBGF sample (dashed-line in Fig. 1) suggested that 79 ps/nm/km is achieved around 1603 nm. We measured CD on a short piece (3.5 m) of the HC-PBGF using interferometric technique [1], (solid-line in Fig. 1), which showed CD of 79 ps/nm/km occurring at 1607 nm. The transmission loss of the fiber at this wavelength is 8.6 dB/km.

As we show below (to the best of our knowledge for the first time), the OEO setup itself offers a method for measuring CD of the whole length of the hollow core fibre. By varying the laser wavelength, the propagation delay through the hollow core fibre changes due to CD, which in turn changes the OEO carrier frequency and cavity free spectral range (FSR). The change of propagation delay can be calculated according to (1) and in this case the subscripts 1 and 2 corresponds to two wavelengths.

We measured the change of OEO carrier frequency (f_drift_) by slightly changing the laser wavelength (less than 1 nm) and repeated this over the spectral range of 1520 nm and 1616 nm. Subsequently, we calculated the change of delay, Δτ according to (1) (FSR of the OEO cavity was measured to be 260 kHz, f_osc_=10 GHz).

| $CD=\frac{\Delta\tau}{\Delta\lambda L}=\frac{1}{\Delta\lambda L}\left[ \frac{f_{drift}}{f_{osc}FSR} \right]$. | (i) |
| --- | --- |

The CD was then calculated according to (i) by dividing Δτ by the change of wavelength ($\Delta\lambda$) and the length of the fiber, L. The results are shown by the diamond symbols in Fig. 1.


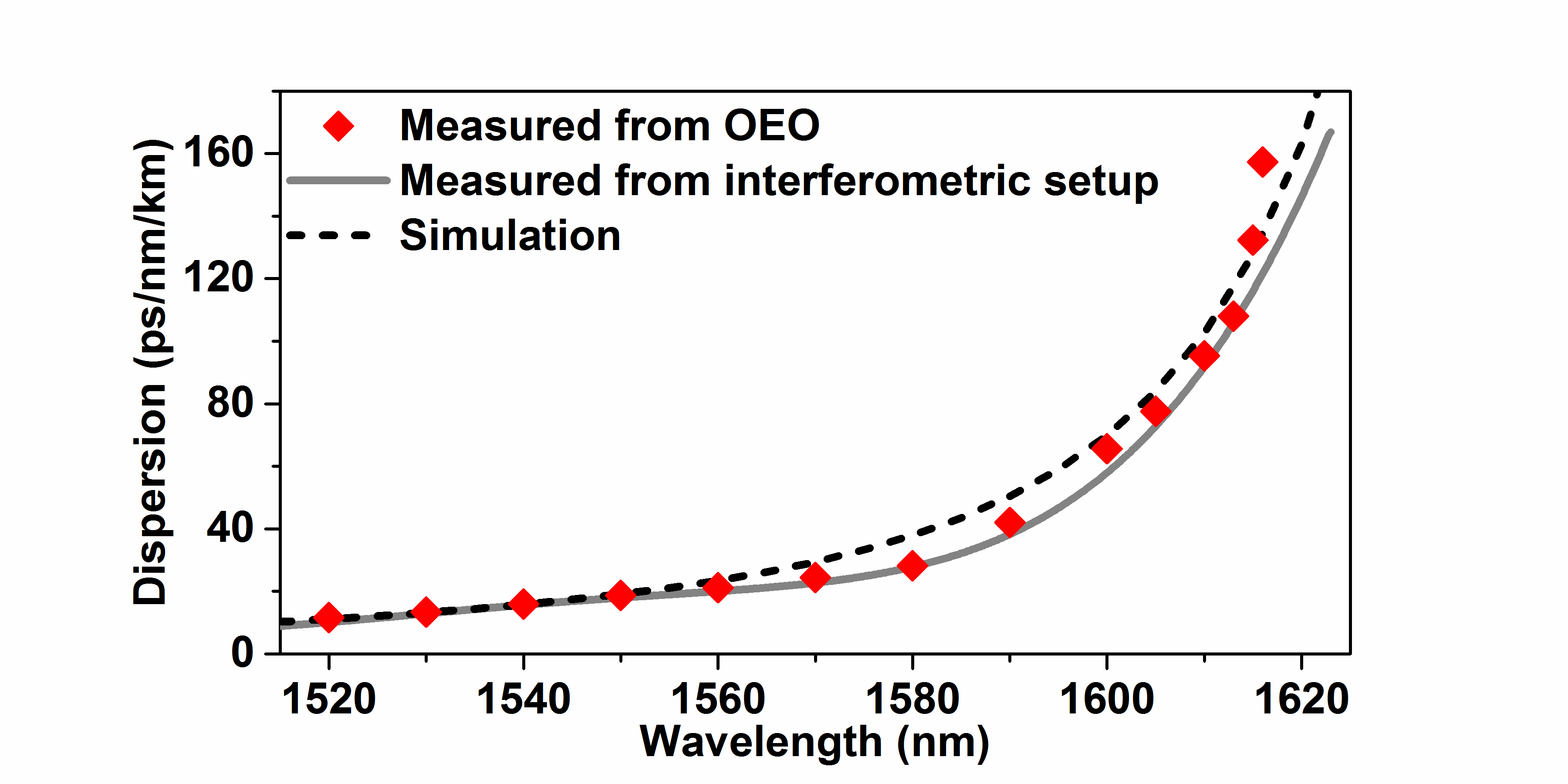


Fig. 1. Chromatic dispersion of the used HC-PBGF.

In Fig. 1 we see that the results meausred on a short piece and entire fibre sample length give very similar result, suggesting good uniformity of the >1 km long HC-PBGF sample (since CD of HC-PBGF depends on its structural parameters). We also conclude that the meaured data is very close to that predicted theoretically, further supporting good agreement between the predicted and measured results shown in the main article text.

[1] Ref. 6 from the main article.
